# Distinct adaptations of muscle endurance but not strength or hypertrophy to low‐load resistance training with and without blood flow restriction

**DOI:** 10.1113/EP091310

**Published:** 2024-03-19

**Authors:** Akito Ida, Kazushige Sasaki

**Affiliations:** ^1^ Department of Life Sciences, Graduate School of Arts and Sciences The University of Tokyo Tokyo Japan

**Keywords:** chronic adaptations, electromyography, local muscle endurance, near‐infrared spectroscopy, strength training

## Abstract

Low‐load resistance training promotes muscle strength and hypertrophic adaptations when combined with blood flow restriction (BFR). However, the effect of BFR on muscle endurance remains unclear. The aim of this study was to clarify the effects of BFR on muscle performance and adaptation, with special reference to local muscle endurance. In experiment 1, eight healthy men performed unilateral elbow flexion exercise to failure at 30% of one‐repetition maximum with BFR (at 40% of estimated arterial occlusion pressure) and free blood flow (FBF). During the exercise, muscle activity and tissue oxygenation were measured from the biceps brachii. In experiment 2, another eight healthy men completed 6 weeks of elbow flexion training with BFR and FBF. The number of repetitions to failure at submaximal load (*R*_max_), the estimated time for peak torque output to decay by 50% during repetitive maximum voluntary contractions (half‐time), one‐repetition maximum, isometric strength and muscle thickness of elbow flexors were measured pre‐ and post‐training. Blood flow restriction resulted in fewer repetitions and lower muscle tissue oxygenation at the end of exercise than FBF, while the muscle activity increased similarly to repetition failure. Blood flow restriction also resulted in a smaller post‐training *R*_max_, which was strongly correlated with the total exercise volume over the 6 week period. Despite the smaller exercise volume, BFR resulted in similar improvements in half‐time, muscle strength and thickness compared with FBF. These results suggest that the application of BFR can attenuate muscle endurance adaptations to low‐load resistance training by decreasing the number of repetitions during exercise, both acutely and chronically.

## Abstract (highlights)

- **What is the central question of this study?**
  How does blood flow restriction (BFR) affect adaptations of muscle endurance, strength and hypertrophy in response to low‐load resistance exercise to volitional failure?
- **What is the main finding and its importance?**
  Low‐load resistance training with and without BFR improved muscle strength and thickness to a similar extent. However, the number of repetitions achieved during submaximal exercise, which is an indicator of muscle endurance, showed a much smaller improvement when trained with BFR than without. The results suggest that BFR can attenuate the improvement in muscle endurance by decreasing the total exercise volume achieved for a certain period of training.

## 1 INTRODUCTION

It is widely known that resistance training improves muscle mass and function. The American College of Sports Medicine (ACSM) guideline recommends using a load of ≥60% of one‐repetition maximum (1RM) with ≤12 repetitions to improve muscle strength, and ≤50% of 1RM with 10–25 repetitions or more to improve local muscle endurance (ACSM, 2009; Deschenes & Garber, 2018). Severe metabolic stress and fatigue typically induced by low‐load, high‐repetition exercise are thought to be an important stimulus for muscle adaptations necessary to improve muscle endurance (ACSM, 2009; Mang et al., 2022). Low‐load resistance exercise with blood flow restriction (BFR), in which an elastic band or a pneumatic cuff placed at the proximal end of the limb partly restricts arterial inflow and almost completely restricts venous outflow from working muscles, has been shown to increase accumulation of metabolites (Loenneke et al., 2011; Suga et al., 2012), gene expression associated with angiogenesis (Ferguson et al., 2018; Larkin et al., 2012; Takano et al., 2005) and mitochondrial protein synthesis (Groennebaek et al., 2018). Chronic adaptations to exercise training with BFR have also been reported, including improvements in mitochondrial respiration (Groennebaek et al., 2018), capillary density (Bjørnsen et al., 2019) and muscle oxidative capacity (Sundberg et al., 1993), all of which can be advantageous for reducing muscle fatigue and maintaining force production.

One feature of exercise with BFR potentially related to muscle and performance adaptations is the reduction in time to task failure or the number of repetitions in comparison to exercise without BFR (Farup et al., 2015; Kolind et al., 2023; Wernbom et al., 2009), resulting in time‐efficient improvements in muscle mass and strength. However, the exercise volume (load × number of repetitions) is considered to play a major role in improving muscle endurance (Campos et al., 2002; Stone & Coulter, 1994). The reduction in exercise volume might outweigh the above‐mentioned benefit from the severe metabolic stress associated with BFR, negatively affecting muscle endurance adaptations. To our knowledge, however, the impact of BFR on exercise volume, muscle endurance adaptations and their association has not been studied systematically. Given that muscle endurance is classified as a health‐related physical fitness component (Pate, 1983), exploring an effective way to induce muscle endurance adaptations might have important implications for maintaining and promoting health, in addition to competitive sports.

Muscle endurance is a broad term that refers to the ability of a muscle or group of muscles to exert external forces repeatedly over a period of time (Vaara et al., 2012). It has been assessed practically using two different measures: (1) the time to task failure or the maximum number of repetitions during low‐intensity submaximal contractions; and (2) the reduction in performance (e.g., peak torque output) during maximal or near‐maximal contractions (Avin & Law, 2011). Although resistance exercise training has been shown to improve both measures (Ebben et al., 2004; Kemmler et al., 2004), submaximal and maximal contractions should differ in energy metabolism, metabolite accumulation and motor unit recruitment. These differences imply that the factors limiting endurance performance are different between submaximal and maximal contractions.

The purpose of this study was to clarify the effects of BFR on muscle performance and adaptation, with special reference to muscle endurance. For this purpose, two experiments were carried out. First, we determined which factors were related to the assumed reduction in exercise volume (i.e., the decrease in the number of repetitions) caused by BFR, examining some indicators of voluntary effort, neuromuscular fatigue and oxygen supply to the muscle (experiment 1). Second, we conducted a 6 week training intervention with and without BFR, associating the acute and chronic effects of BFR on muscle endurance (experiment 2). We hypothesized that the decrease in exercise volume attributable to BFR might attenuate the training‐induced improvement in muscle endurance performance during submaximal but not maximal contractions.

## 2 MATERIALS AND METHODS

### 2.1 Ethical approval

This study was performed in line with the principles of the *Declaration of Helsinki* without being registered. Approval was granted by the Ethical Review Committee for Experimental Research involving Human Subjects, the Graduate School of Arts and Sciences, the University of Tokyo (no. 797). All the participants were informed of the procedures and possible risks and gave their informed consent prior to the experiment.

### 2.2 Participants

We recruited healthy men aged 18–50 years, for whom the exclusion criteria were as follows: resting systolic/diastolic blood pressure ≥140/90 mmHg, 1RM of unilateral elbow flexion <6 kg (to ensure that the exercise load is equal to or above the minimum in our equipment) and having an exercise habit (only for experiment 2). The sample size in experiment 1 was determined by the following procedure. Initially, we estimated an effect size (Cohen's *d*) in Student's paired *t*‐test to be 1.5 from previous studies investigating the acute effect of BFR on the maximum number of repetitions (Farup et al., 2015; Kolind et al., 2023; Wernbom et al., 2009). Using power analysis software (G*Power v.3.1.9.6, University of Düsseldorf, Germany), the minimal sample size required for an 80% statistical power and an α error of 5% (two tailed) resulted in seven participants. To allow for dropout, the sample size was increased to eight. This sample size is comparable to those of previous studies investigating the chronic effects of BFR training with the upper limbs (Dankel et al., 2016). The same sample size was used in experiment 2 because we assumed that the change in the number of repetitions attributable to BFR in each exercise session has a cumulative effect on the training‐induced muscle adaptation. As a result, eight healthy right‐handed males (26.9 ± 6.6 years of age, 178.3 ± 6.2 cm tall and weighing 78.5 ± 9.9 kg) participated in experiment 1, and another eight healthy right‐handed males (20.8 ± 3.1 years of age, 170.3 ± 3.6 cm tall and weighing 58.0 ± 5.7 kg) participated in experiment 2.

### 2.3 Procedures

#### 2.3.1 Exercise

Unilateral elbow flexion exercise with a plate‐loaded dumbbell (SDB‐1, IVANKO Barbell Company, USA) was used as an exercise model for both experiments 1 and 2. During exercise, participants sat on a preacher curl bench (GPCB329, Body‐Solid, USA) with an arm pad inclined 30° from the vertical. With the aid of a metronome, concentric and eccentric actions were performed rhythmically for 1 s each, with a full range of motion (∼0–120° of elbow flexion). The exercise load was set close to 30% of 1RM using a combination of weight plates (SDRUB, IVANKO Barbell Company, USA) of 0.5, 1.25, 2.5 and 5 kg. The testing protocol of 1RM was the same as that of a previous study (Ball & Scurr, 2010). Participants performed the exercise until they could no longer lift the load with the predetermined cadence (Kolind et al., 2023). Verbal encouragement was given throughout the exercise.

#### 2.3.2 Application of blood flow restriction

Blood flow restriction was applied by wrapping a 5‐cm‐wide elastic band specially designed for BFR training (Kaatsu Master Mini, Sato Sports Plaza, Japan) around the proximal part of the upper arm. The pressure was set at 40% of the arterial occlusion pressure estimated from the upper arm circumference and resting blood pressure using the following formula (Loenneke et al., 2015): arterial occlusion pressure (in millimetres of mercury) = 0.514 × systolic blood pressure + 0.339 × diastolic blood pressure + 1.461 × arm circumference (in centimetres) + 17.236. The use of higher pressure was considered unsuitable for improving muscle endurance because the number of repetitions achieved during elbow flexion exercise could be reduced substantially with increasing the occlusion pressure (Counts et al., 2016).

### 2.4 Experiment 1

In accordance with previous studies examining the acute effects of BFR (Farup et al., 2015; Kolind et al., 2023; Wernbom et al., 2009), we used a within‐participant crossover design. The exercise was performed with the right arm, except for one participant who had a history of injury in the right arm. Each participant visited the laboratory three times. We instructed participants to refrain from consuming alcohol and caffeine for 24 h before the experiment. During the initial visit, 1RM of unilateral elbow flexion, resting blood pressure and upper‐arm circumference were measured. The participants then performed 20 repetitions of elbow flexion at 30% of 1RM with BFR and, subsequently, 20 repetitions with free blood flow (FBF) for practice. Next, the participants were scheduled for the two testing visits to be conducted at the same time of day, ≥5 days apart.

During the second and third visits, they performed the unilateral elbow flexion exercise with either BFR or FBF after the re‐evaluation of 1RM. There was a rest of ≥5 min between the 1RM measurement and the exercise. The order of exercise conditions was randomized and counterbalanced across participants.

During the exercise, muscle activity and tissue oxygenation were recorded from the biceps brachii muscle by using surface EMG and near‐infrared spectroscopy, respectively. Bipolar electrodes with an inter‐electrode distance of 12 mm (DL‐141, S&ME, Japan) were placed on the short head of the biceps brachii, two‐thirds of the distance from the medial acromion to the antecubital fossa (Hermens et al., 1999). An earth electrode was placed on the medial clavicle. Before electrode placement, the skin was shaved (if needed), abraded, and cleaned with alcohol wipes. The EMG signals were amplified (gain: ×400), band‐pass filtered (10–500 Hz), and sampled at a rate of 2 kHz using a data acquisition system (PowerLab/16SP, ADInstruments, New Zealand). For the recording of tissue oxygenation, we used a small, spatially resolved near‐infrared spectroscopy device (Oxy‐Pro, Astem, Japan). The measurement characteristics of its prototype were analysed previously using Monte Carlo simulation and a phantom experiment (Niwayama & Unno, 2021). Optical probes consisting of light‐emitting diodes with wavelengths of 770 and 830 nm and photodetectors located 20 and 30 mm from the light source were placed on the long head of the biceps brachii, two‐thirds of the distance from the medial acromion to the antecubital fossa (Kojima et al., 2020). We used surgical tape to secure the device and a light‐shielding band to minimize the influence of ambient light. The oxygenated and deoxygenated haemoglobin (Hb) data were obtained after correcting for the thickness of the fat layer (Niwayama et al., 2000) measured with a B‐mode ultrasound device (MiruCube II, Global Health, Japan). The tissue oxygen saturation ($\mathrm{St}O_{2}$), a ratio of oxygenated to total Hb (the sum of oxygenated and deoxygenated Hb) concentrations, was monitored continuously and recorded at a sampling rate of 10 Hz. Immediately after termination of the exercise, the rating of perceived exertion (RPE) was measured with a 0–10 category scale. This scale is considered more useful than the 6–20 Borg scale for assessing the intensity of resistance exercise (Hackett et al., 2012).

### 2.5 Experiment 2

In accordance with previous studies examining the effects of training with BFR on muscle adaptations (Counts et al., 2016; Farup et al., 2015), we used a within‐participant unilateral model, with each training condition assigned to either the left or the right arm. The two conditions were counterbalanced by arm dominance (half of the participants were assigned to BFR with the dominant arm and vice versa) and by the maximal number of repetitions achieved during submaximal elbow flexions (*R*_max_; see below) at pre‐intervention measurement. The order of BFR and FBF conditions was alternated for each exercise session. There was a rest of ≥10 min between the BFR and FBF conditions.

Experiment 2 consisted of a 6 week training intervention (Bjørnsen et al., 2019; Farup et al., 2015; Groennebaek et al., 2018) and pre‐ and post‐intervention measurements of muscle endurance, muscle strength, muscle thickness and echo intensity. We instructed participants to maintain their regular diet during the experimental period. The pre‐intervention measurements involved three visits, and the post‐intervention measurements involved two visits, with an interval of 2–5 days between each visit. Participants performed exercise training twice a week during the intervention period (at intervals of ≥72 h). This is based on the ACSM position statement, which recommends 2–3 days per week as the training frequency for gaining muscle mass and endurance for novice participants (ACSM, 2009).

**FIGURE 1:**
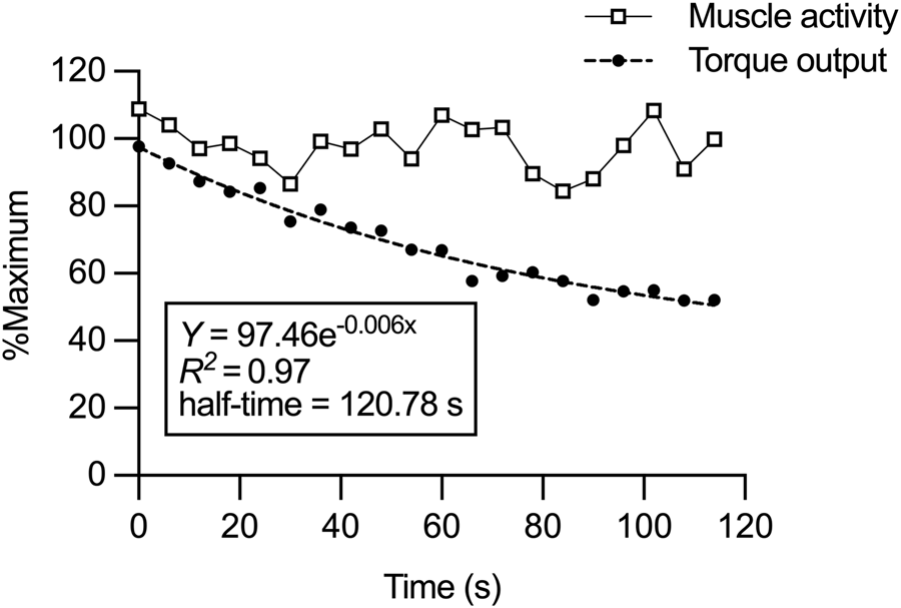
Representative data of peak torque output and the biceps brachii muscle activity (both averaged for every three contractions) plotted against time during a series of maximal voluntary isometric elbow flexions without blood flow restriction. Both the peak torque output and the muscle activity were normalized to their respective maximal values recorded during the isometric strength test. An exponential function was used to estimate the half‐time (i.e., the time for peak torque output to decay by 50%).

We used two different measures of muscle endurance (note that both these measurements were always performed without restricting blood flow). One measure was *R*_max_, representing the number of repetitions to failure during the unilateral elbow flexion exercise. The same load (30% of 1RM determined before intervention) was used for pre‐ and post‐intervention measurements of *R*_max_. The other measure was half‐time (Figure 1), defined as the estimated time for peak torque output to decay by 50% during a series of maximum voluntary isometric contractions (MVCs). The protocol for half‐time measurement consisted of 60 MVCs performed on a dynamometer (AO‐500N, Applied Office, Japan) with a cadence of one ballistic contraction every 2 s (Huczel & Clarke, 1992), when the participants were instructed to contract the elbow flexor muscles as hard as they could. During the measurement, the participants sat on a chair, with the shoulder abducted to 90°, the elbow flexed to 90° and the forearm supinated to 90°. To ensure an isometric contraction, the wrist and the upper arm were secured with a metal cuff and an inelastic strap, respectively. Elbow flexion torque was sampled at 2 kHz using the data acquisition system. A real‐time torque output and the maximal torque achieved during an isometric strength test (described below) were displayed on an oscilloscope (SS‐7604, Iwatsu Electric, Japan) to provide visual feedback to the participants. During the measurement, surface EMG was recorded from the biceps brachii muscle in a manner similar to that of experiment 1.

We measured both dynamic (1RM) and static (isometric torque) muscle strengths. The protocol of 1RM testing was the same as in experiment 1. The isometric strength of elbow flexor muscles was measured as the peak torque applied to the dynamometer. The participants were instructed to produce the elbow flexion torque as strongly as possible for 3 s. Measurements were taken three times, with a 3 min rest between each, prior to the measurement of half‐time. The highest value was used for further analysis.

Muscle thickness and echo intensity of elbow flexors were measured using the same ultrasound device as used in experiment 1. The measurement site was at 60% of the upper arm length (i.e., the distance from the acromion process of the scapular to the lateral epicondyle of the humerus; Miyatani et al., 2004). During the measurement, the participants stood in an upright position, with the elbow joint fully extended and the forearm supinated to 90°. A transducer with a 6 MHz scanning head coated with water‐soluble transmission gel was placed perpendicular to the underlying muscle and bone tissues. The muscle thickness was defined as the distance from the border of adipose and muscle tissues including the biceps brachii and the brachialis to the upper edge of the humerus, which showed a strong correlation with the muscle volume measured by MRI (Miyatani et al., 2004). The echo intensity was determined by 8‐bit grey‐scale analysis (0, black; 256, white) of the transverse ultrasound images using the standard histogram function in ImageJ (v.1.46r; National Institutes of Health, USA). A region of interest was selected as a rectangle between the uppermost part of the bone echo of the humerus and the superficial fascia of the biceps brachii, with a fixed width of 10 mm.

### 2.6 Data analysis

In experiment 1, the number of repetitions, RPE, EMG data and tissue oxygenation data were analysed. For the EMG data, root‐mean‐square amplitude and mean power frequency were calculated over five repetitions at the beginning (First5), in the middle (Mid5) and at the end (Last5) of the exercise. The root‐mean‐square amplitude for each repetition (2 s time window) was normalized to that obtained from the 1RM trial (the highest 1 s) and used as an indicator of muscle activity. For the calculation of mean power frequency, fast Fourier transform with a Hamming window function was applied to a series of 0.256 s segments (frequency resolution, 3.91 Hz) overlapping each other by 50%. We did not separate the data into concentric and eccentric phases because the fatigue‐induced reduction in mean power frequency was observed in the biceps brachii, regardless of contraction mode (Potvin, 1997). The tissue oxygenation data ($\mathrm{St}O_{2}$, oxygenated‐Hb, deoxygenated‐Hb and total‐Hb) were synchronized with the EMG data and averaged over First5, Mid5 and Last5.

In experiment 2, total exercise volume was obtained by multiplying the load and the total number of repetitions over the training period. For the measurement of half‐time, the peak torque output and the biceps brachii muscle activity were analysed for each contraction. The peak torque output was averaged for every three contractions (Figure 1). The averaged torque output was then regressed against time using an exponential function, from which the half‐time was calculated (Huczel & Clarke, 1992). To confirm that the decrease in peak torque with time was attributable mainly to peripheral muscle fatigue, the muscle activity quantified as the root‐mean‐square amplitude for the highest 0.5 s was normalized to that recorded during the isometric strength test and averaged over the 60 MVCs.

### 2.7 Statistical analysis

Statistical analysis was performed using GraphPad Prism 9 software (GraphPad Software, USA). For all variables, data normality was assessed using the Kolmogorov–Smirnov test. The data with non‐normal distribution were logarithmically transformed. As a result, the data followed a normal or log‐normal distribution in all the variables and thus are expressed as the mean and SD. In experiment 1, Student's paired *t*‐test was used to determine the effect of BFR on the number of repetitions to failure and RPE. Within‐participant two‐way ANOVA (condition × time) was used to determine the effects of BFR on the changes in EMG and tissue oxygenation data during the exercise. In experiment 2, Student's paired *t*‐test was used to determine the effect of BFR on the total exercise volume. Within‐participant two‐way ANOVA (condition × time) was used to determine the effect of BFR on *R*_max_, half‐time, average muscle activity during half‐time measurement, 1RM, maximum isometric torque, muscle thickness and echo intensity. When a significant interaction was found, post‐hoc multiple comparisons were conducted using Student's paired *t*‐test, with the false discovery rate method (Benjamini & Hochberg, 1995) used to correct *P*‐values. Linear regression analysis was performed to determine the association of the percentage changes in *R*_max_ and half‐time with the total exercise volume. A *P*‐value of <0.05 was considered significant. Cohen's effect size (*d*), generalized *η* ^2^ (*η*_G_^2^) and the coefficient of determination (*R* ^2^) were reported as a measure of effect size for Student's paired *t*‐test, within‐participant ANOVA and linear regression analysis, respectively. Using *η*_G_^2^ as a measure of effect size is deemed statistically more suitable for repeated‐measures ANOVA than using partial *η* ^2^ (Olejnik & Algina, 2003).

## 3 RESULTS

### 3.1 Estimated arterial occlusion pressure

The estimated arterial occlusion pressures on the upper arm were 158.2 ± 13.7 and 145.3 ± 8.0 mmHg in experiments 1 and 2, respectively, resulting in target pressures during exercise to be 63 ± 6 and 58 ± 3 mmHg, respectively.

### 3.2 Experiment 1

#### 3.2.1 Number of repetitions and rating of perceived exertion

**FIGURE 2:**
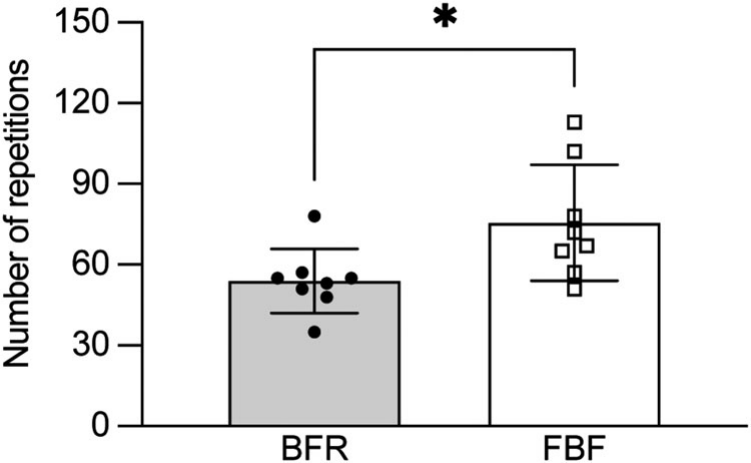
Number of repetitions to failure during unilateral elbow flexion exercise at 30% of one‐repetition maximum with blood flow restriction (BFR) or free blood flow (FBF). Values represent means and SD with individual data (*n* = 8 for each). ^*^ *P* < 0.05 by Student's paired *t*‐test.

Figure 2 shows the number of repetitions to failure during the exercise with BFR and FBF. The exercise with BFR resulted in significantly fewer repetitions than that with FBF (−26.0 ± 16.4%, *P* = 0.013, *d* = 1.158). In contrast, RPE evaluated immediately after the termination of exercise was not significantly different: 8.5 ± 0.8 and 8.0 ± 0.9 for the exercise with BFR and FBF, respectively (*P* = 0.231, *d* = 0.540).

#### 3.2.2 Muscle activity

**FIGURE 3:**
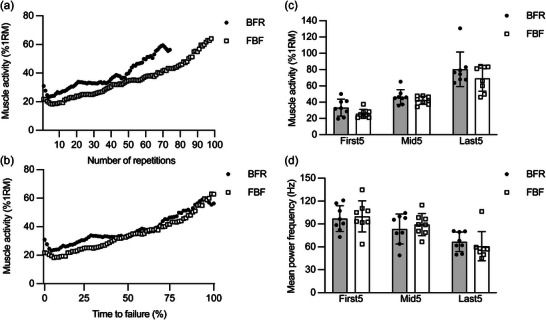
Changes in EMG data of the biceps brachii muscle during unilateral elbow flexion exercise at 30% of one‐repetition maximum (1RM) with blood flow restriction (BFR) or free blood flow (FBF). Root‐mean‐square amplitude in each repetition was normalized to that measured during the 1RM trial and used as an indicator of muscle activity. Mean power frequency was calculated by applying fast Fourier transformation to a series of 0.256 s segments (frequency resolution, 3.91 Hz) overlapping each other by 50%. (a) Representative data of the biceps brachii activity from one participant plotted against the number of repetitions. (b) Representative data of the biceps brachii activity from one participant plotted against the normalized time to failure. (c) Differences in the average biceps brachii activity over five repetitions from the beginning of the exercise (First5), right in the middle of the exercise duration (Mid5) and immediately before the task failure (Last5). (d) Differences in the average biceps brachii mean power frequency over First5, Mid5 and Last5. In (c) and (d), values represent means and SD with individual data (*n* = 8 for each).

Figure 3 shows representative and group data of the biceps brachii muscle activity during the exercise. Although the exercise with BFR showed an early rise in muscle activity in comparison to the exercise with FBF (Figure 3a), the difference was much reduced when the exercise duration was normalized to the respective time to failure (Figure 3b). The two‐way ANOVA on the group data (Figure 3c) revealed a significant main effect of time (*P* < 0.001, *η*_G_^2^ = 0.720), but not a main effect of condition (*P* = 0.136, *η*_G_^2^ = 0.092) or an interaction (*P* = 0.599, *η*_G_^2^ = 0.015). Likewise, the two‐way ANOVA on the group data of the biceps brachii mean power frequency (Figure 3d) revealed a significant main effect of time (*P* < 0.001, *η*_G_^2^ = 0.438), but not a main effect of condition (*P* = 0.760, *η*_G_^2^ = 0.001) or an interaction (*P* = 0.241, *η*_G_^2^ = 0.022).

#### 3.2.3 Muscle tissue oxygenation

**FIGURE 4:**
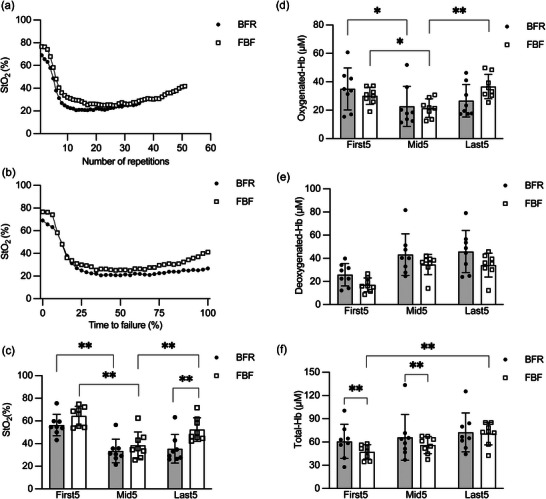
Changes in the concentration of oxygenated, deoxygenated and total haemoglobin (Hb) of the biceps brachii muscle tissue during unilateral elbow flexion exercise at 30% of one‐repetition maximum with blood flow restriction (BFR) or free blood flow (FBF). (a) Representative data from one participant plotted against the number of repetitions. (b) Representative data from one participant plotted against the normalized time to failure. (c) Differences in the average tissue oxygen saturation ($\mathrm{St}O_{2}$) over five repetitions from the beginning of the exercise (First5), right in the middle of the exercise duration (Mid5) and immediately before the task failure (Last5). (d) Differences in the average oxygenated Hb concentration over First5, Mid5 and Last5. (e) Differences in the average deoxygenated Hb concentration over First5, Mid5 and Last5. (f) Differences in the average total Hb concentration over First5, Mid5 and Last5. In (c–f), values represent means and SD with individual data (*n* = 8 for each). ^*^Adjusted *P* < 0.05 and ^**^adjusted *P* < 0.01 by Student's paired *t*‐test with the false discovery rate method.

Figure 4 shows representative and group data of the biceps brachii muscle tissue oxygenation during the exercise. Typically, the exercise caused a drop in $\mathrm{St}O_{2}$ within the first quarter of exercise in both BFR and FBF (Figure 4a). The exercise with FBF showed a slight recovery in $\mathrm{St}O_{2}$ during the last quarter of exercise, whereas the exercise with BFR did not (Figure 4b). The two‐way ANOVA on the group data of $\mathrm{St}O_{2}$ (Figure 4c) revealed significant main effects of time (*P* < 0.001, *η*_G_^2^ = 0.514) and condition (*P* < 0.001, *η*_G_^2^ = 0.184) and a significant interaction (*P* < 0.001, *η*_G_^2^ = 0.058). The post‐hoc tests revealed significant differences in $\mathrm{St}O_{2}$ between First5 and Mid5 in both conditions (BFR: adjusted *P* < 0.001, *d* ^^= 3.151; FBF: adjusted *P* = 0.001, *d* ^^= 2.132), between Mid5 and Last5 in FBF (adjusted *P* < 0.001, *d* = 4.845) and between conditions at Last5 (adjusted *P* < 0.001, *d *= 2.832). The two‐way ANOVA on the group data for oxygenated Hb (Figure 4d) revealed a significant main effect of time (*P* < 0.001, *η*_G_^2^ = 0.180) and a significant interaction (*P* < 0.001, *η*_G_^2^ = 0.089), but not a main effect of condition (*P* = 0.832, *η*_G_^2^ = 0.004). The post‐hoc tests revealed significant differences in oxygenated Hb between First5 and Mid5 in both conditions (BFR: adjusted *P* = 0.004, *d* ^^= 1.582; FBF: adjusted *P* = 0.007, *d* ^^= 1.461) and between Mid5 and Last5 in FBF (adjusted *P* < 0.001, *d* = 3.590). The two‐way ANOVA on the group data of deoxygenated Hb (Figure 4e) revealed a significant main effect of time (*P* < 0.001, *η*_G_^2^ = 0.339), but not a main effect of condition (*P* = 0.056, *η*_G_^2^ = 0.143) or an interaction (*P* = 0.547, *η*_G_^2^ = 0.004). The two‐way ANOVA on the group data of total Hb (Figure 4f) revealed a significant main effect of time (*P* < 0.001, *η*_G_^2^ = 0131) and a significant interaction (*P* < 0.001, *η*_G_^2^ = 0.018), but not a main effect of condition (*P* = 0.422, *η*_G_^2^ = 0.048). The post‐hoc tests revealed a significant difference in total Hb between Mid5 and Last5 in FBF (adjusted *P* < 0.001, *d* ^^= 2.49).

### 3.3 Experiment 2

#### 3.3.1 Number of repetitions in each session and total exercise volume

**FIGURE 5:**
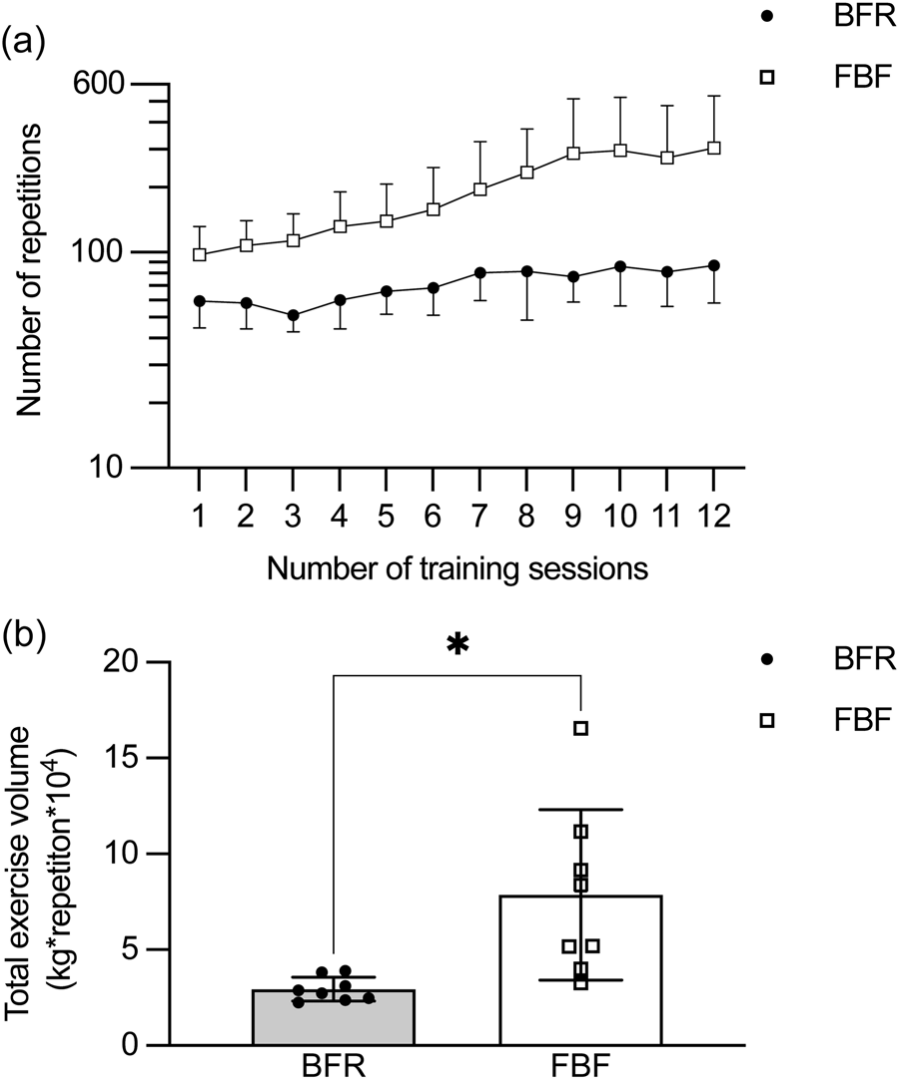
Changes in the number of repetitions to failure in each exercise session (a) and total exercise volume over 6 weeks (b) of unilateral elbow flexion exercise training at 30% of one‐repetition maximum with blood flow restriction (BFR) or free blood flow (FBF). In (a), the number of repetitions is plotted in logarithmic scale for clarity. Values represent means and SD (*n* = 8 for each). In (b), values represent means and SD with individual data (*n* = 8 for each). ^*^ *P* < 0.05 by Student's paired *t*‐test.

Figure 5 shows changes in the number of repetitions and the total exercise volume (load × repetitions) during the 6 week training period. As shown in Figure 5a, the number of repetitions to failure in each exercise session increased progressively over the training period (main effect of time: *P* < 0.001, *η*_G_^2^ = 0.171) but was consistently lower in the exercise with BFR than with FBF (main effect of condition: *P* = 0.018, *η*_G_^2^ = 0.289). Student's paired *t*‐test revealed that the total exercise volume was significantly lower in BFR than in FBF (−52.5 ± 23.6%, *P* = 0.016, *d* = 1.110; Figure 5b).

#### 3.3.2 Muscle endurance

**FIGURE 6:**
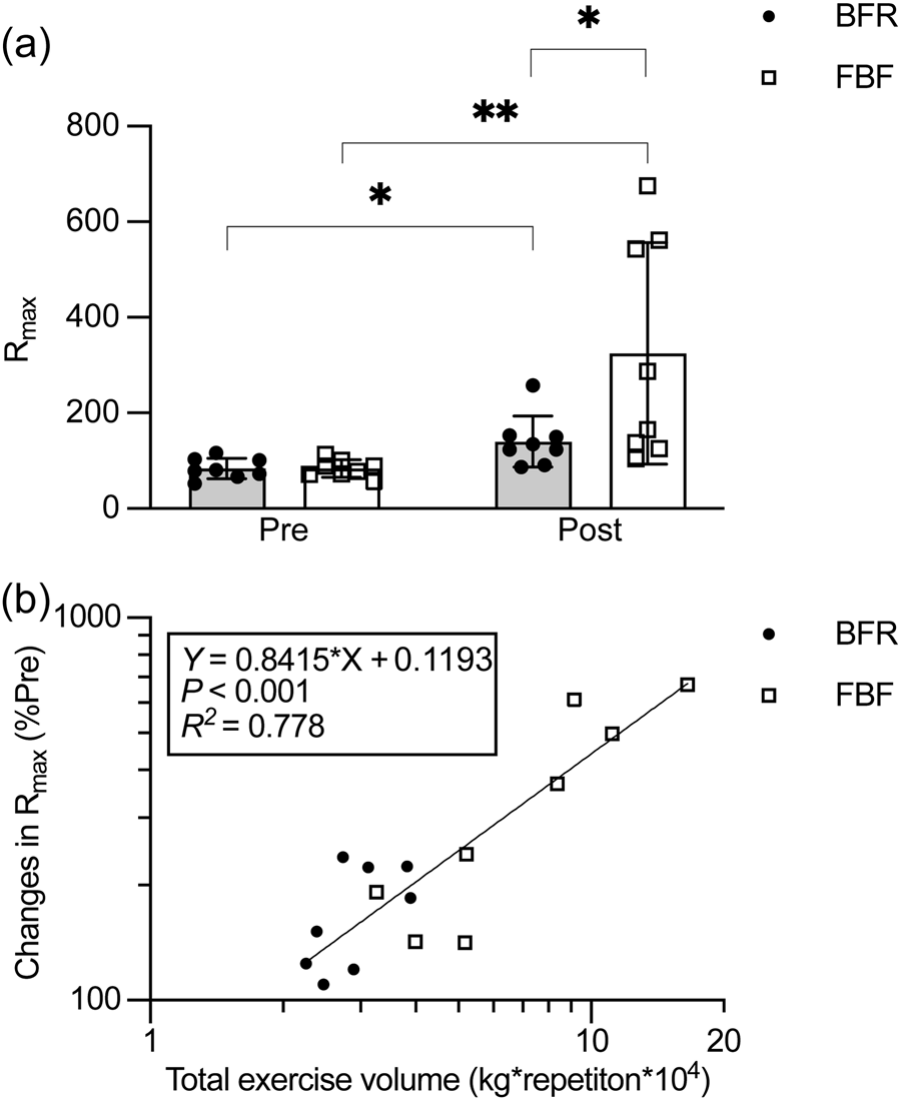
Changes in the number of repetitions (*R*_max_) achieved in a bout of elbow flexion exercise at 30% of one‐repetition maximum before (Pre) and after (Post) a 6 week training period (a) and the association of the percentage changes in *R*_max_ with the total exercise volume (b). The exercise training was performed with blood flow restriction (BFR) or free blood flow (FBF), whereas the pre‐ and post‐intervention measurements of *R*_max_ were performed consistently without restricting blood flow. The data from the BFR and FBF conditions were combined before linear regression analysis. The total exercise volume and percentage changes in *R*_max_ were logarithmically transformed to account for non‐normal distribution. In (a), values represent means and SD with individual data (*n* = 8 for each). ^*^Adjusted *P* < 0.05 and ^**^adjusted *P* < 0.01 by Student's paired *t*‐test with the false discovery rate method. In (b), raw percentage changes in *R*_max_ and total exercise volume data are plotted in logarithmic scale for clarity, whereas the linear regression equation is derived from the log‐transformed data.

Figure 6 shows the changes in *R*_max_ before and after the training period. The two‐way ANOVA revealed significant main effects and a significant interaction of time and condition (main effect of time: *P *= 0.010, *η*_G_^2^ = 0.305; main effect of condition: *P *= 0.037, *η*_G_^2^ = 0.145; and interaction: *P *= 0.041, *η*_G_^2^ = 0.146). As shown in Figure 6a, *R*_max_ was significantly improved after training with BFR (69.5 ± 51.5%, adjusted *P* = 0.011, *d* = 1.285) and FBF (263.0 ± 208.4%, adjusted *P* = 0.001, *d* = 1.803). However, the extent of improvement differed significantly between conditions, with the value being higher in FBF than in BFR at post‐training (adjusted *P* = 0.036, *d* = 1.084). When BFR and FBF data were combined, a strong positive association (*P* < 0.001, *R^2^* = 0.778) was observed between the percentage change in *R*_max_ and the total exercise volume (Figure 6b).

**FIGURE 7:**
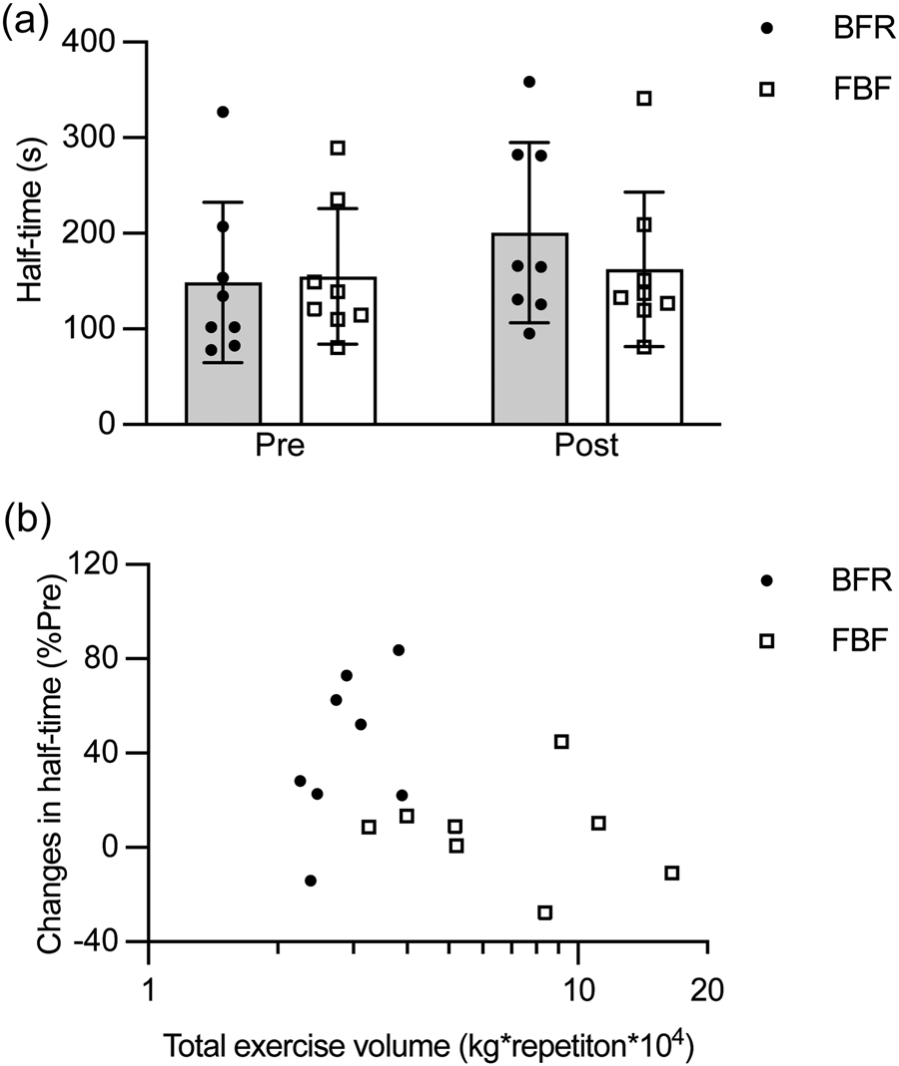
Changes in the time for peak torque output to decay by 50% (half‐time) estimated from a series of maximum voluntary isometric contractions (MVCs) of elbow flexors before (Pre) and after (Post) a 6 week training period (a) and the association of the percentage changes in half‐time with the total exercise volume (b). The exercise training was performed with blood flow restriction (BFR) or free blood flow (FBF), whereas the pre‐ and post‐intervention measurements of half‐time were performed consistently without restricting blood flow. The data from the BFR and FBF conditions were combined before linear regression analysis. The total exercise volume was logarithmically transformed to account for non‐normal distribution. In (a), values represent means and SD with individual data (*n* = 8 for each). In (b), raw total exercise volume data are plotted in logarithmic scale for clarity.

Figure 7a shows the changes in half‐time (the estimated time for peak torque output to decay by 50% during a series of MVCs) before and after the training period. The two‐way ANOVA revealed a significant main effect of time (*P *= 0.002, *η*_G_^2^ = 0.036), but not a main effect of condition (*P *= 0.430, *η*_G_^2^ = 0.010) or an interaction (*P *= 0.285, *η*_G_^2^ = 0.020). These results indicate no significant difference between BFR and FBF conditions in the improvement in half‐time (BFR, 41.3 ± 32.4%; FBF, 6.1 ± 20.9%) after training. When BFR and FBF data were combined, there was no significant association (*P* = 0.134, *R* ^2^ = 0.153) of the percentage change in half‐time with the total exercise volume (Figure 7b). For the averaged muscle activity during the measurement of half‐time, the two‐way ANOVA revealed no significant main effect (main effect of time: *P *= 0.981, *η*_G_^2^ < 0.001; main effect of condition: *P *= 0.287, *η*_G_^2^ = 0.036) or interaction (*P *= 0.127, *η*_G_^2^ = 0.046).

#### 3.3.3 Muscle strength, thickness and echo intensity

**FIGURE 8:**
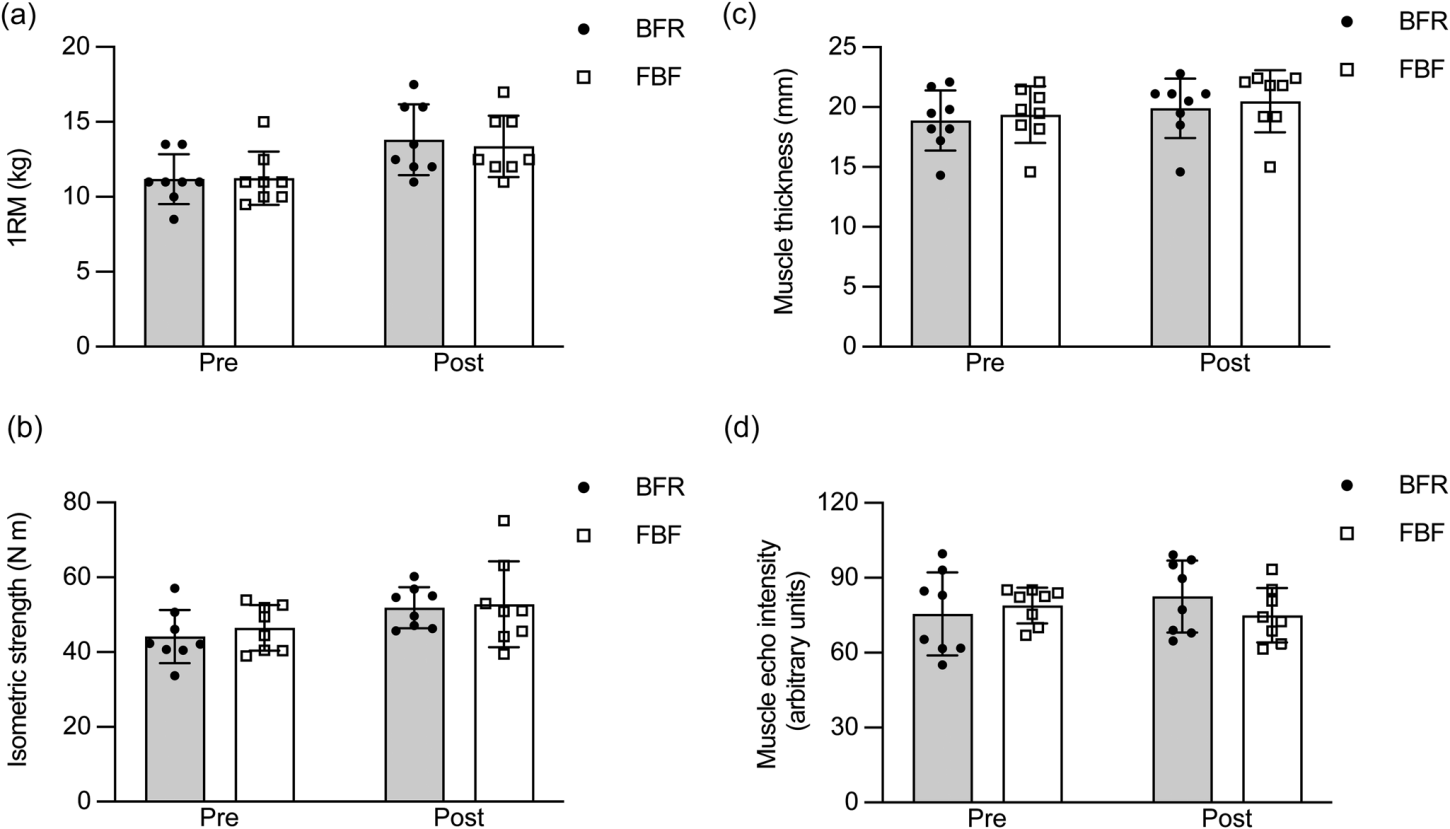
Changes in one‐repetition maximum (1RM; a), isometric strength (b), muscle thickness (c) and echo intensity (d) of elbow flexors before (Pre) and after (Post) 6 weeks of unilateral elbow flexion exercise training with blood flow restriction (BFR) or free blood flow (FBF). Values represent means and SD with individual data (*n* = 8 for each).

Figure 8 shows the changes in muscle strengths, thickness and echo intensity of elbow flexors before and after the training period. The two‐way ANOVA for 1RM (Figure 8a) revealed a significant main effect of time (*P* < 0.001, *η*_G_^2^ = 0.290), but not a main effect of condition (*P* = 0.584, *η*_G_^2^ = 0.003) or an interaction (*P* = 0.068, *η*_G_^2^ = 0.005). The two‐way ANOVA for the maximum isometric torque (Figure 8b) revealed a significant main effect of time (*P* = 0.002, *η*_G_^2^ = 0.185), but not a main effect of condition (*P* = 0.564, *η*_G_^2^ = 0.012) or an interaction (*P* = 0.661, *η*_G_^2^ = 0.003). The two‐way ANOVA for the muscle thickness (Figure 8c) revealed a significant main effect of time (*P* = 0.015, *η*_G_^2^ = 0.050), but not a main effect of condition (*P* = 0.116, *η*_G_^2^ = 0.014) or an interaction (*P* = 0.840, *η*_G_^2^ < 0.001). These results indicate no significant difference between BFR and FBF conditions in the improvement in 1RM (BFR, 23.6% ± 11.2%, FBF, 19.2% ± 8.0%), maximum isometric torque (BFR, 19.1% ± 13.7%, FBF, 12.9% ± 12.6%) or muscle thickness (BFR, 5.7% ± 6.1%, FBF, 5.7% ± 4.4%) after training. Figure 8d shows the changes in echo intensity of elbow flexors before and after the training period. The two‐way ANOVA revealed no significant main effect of time (*P* = 0.659, *η*_G_^2^ = 0.007) or condition (*P* = 0.696, *η*_G_^2^ = 0.004). There was also no significant interaction of time and condition (*P* = 0.152, *η*_G_^2^ = 0.049). A significant positive association was observed between the percentage changes in 1RM and muscle thickness (*P* = 0.036, *R* ^2^ = 0.278). In contrast, the percentage change in maximum isometric torque was not significantly associated with the change in muscle thickness (*P* = 0.845, *R* ^2^ = 0.002) or 1RM (*P* = 0.684, *R* ^2^ = 0.012).

## 4 DISCUSSION

The main finding of this study is that although resistance training with both BFR and FBF improved *R*_max_, the training with BFR resulted in less improvement than that with FBF. Application of BFR has been shown to promote muscle hypertrophy and strength gains when combined with low‐load resistance training, but this study is the first to show that BFR can have a negative impact on the improvement of muscle endurance. We also found that the application of BFR resulted in a decrease in exercise volume (i.e., reduced number of repetitions to failure) without changing the subjective effort or muscle activity at termination of the exercise. As illustrated in Figure 5a, the difference in the number of repetitions between BFR and FBF increased gradually during the training period, such that the average number of repetitions in each session was much lower in BFR (71.4 ± 12.3) than in FBF (195.0 ± 80.2). These data agree with those reported by Farup et al. (2015), who also studied the effect of elbow flexion exercise with and without BFR. Furthermore, there was a strong positive association between the increase in *R*_max_ and the total exercise volume over the training period.

In experiment 1, the application of BFR resulted in a reduction of 26.0% ± 16.4% in the number of repetitions to failure (Figure 2). However, BFR had no significant effect on RPE recorded immediately after the termination of exercise, which supports the view that the perceived exertion is a factor limiting endurance performance (Pageaux & Lepers, 2016). Also, BFR had no significant effect on the amplitude (Figure 3c) or frequency (Figure 3d) of the biceps brachii EMG at any time point relative to the exercise duration. Similar results were reported in a recent meta‐analysis (Cerqueira et al., 2022). These data can be interpreted as faster fatigue development in BFR than in FBF, reflecting a greater central motor command needed to compensate for the exacerbated neuromuscular fatigue. It should be noted that the muscle activity remained submaximal (∼70%–80% of 1RM) even at the end of the exercise with and without BFR. This result might be explained not so much by the neural inhibition, but by the underestimation of muscle activity owing to the lack of distinguishing between the concentric and eccentric phases of elbow flexion exercise. The EMG activity is known to be substantially higher in the concentric phase than in the eccentric phase (Sasaki & Ishii, 2016; Wernbom et al., 2009). If we had recorded the joint angle data and excluded the eccentric phases from the analysis, the normalized biceps brachii activity at the end of exercise would have been closer to 100% of 1RM.

In contrast, there was a difference in muscle tissue oxygenation, in which $\mathrm{St}O_{2}$ was lower in BFR than in FBF at the end of exercise (Figure 4c). This result was attributed mainly to the lack of increase in the oxygenated Hb from the middle to the end of the exercise in BFR (Figure 4d). Therefore, it is likely that during the second half of the exercise, compensatory vasodilatation was facilitated in FBF but not in BFR. The findings that BFR influenced the tissue oxygenation but not the muscle activity at the end of exercise are generally consistent with those from a previous study on knee extensors (Kolind et al., 2023), suggesting that BFR accelerates peripheral and central fatigue development and, consequently, reduces the time or number of repetitions to volitional failure.

The difference in muscle tissue oxygenation between BFR and FBF might lead to different exercise volumes and adaptations associated with muscle endurance performance. Specifically, we speculate that BFR impaired mitochondrial adaptation by decreasing the reliance on aerobic metabolism during exercise over the training period. This speculation is supported by recent findings that BFR attenuated both the reactive oxygen species emissions from mitochondria (Petrick et al., 2019) and the improvement in mitochondrial respiratory capacity in the vastus lateralis muscle after 6 weeks of one‐leg squat training (Pignanelli et al., 2020). It is also worth noting that local muscle endurance is limited not only by the mitochondrial capacity but also by oxygen delivery to the muscles. In fact, 4 weeks of low‐load knee extension training, with one leg with BFR and the other leg with FBF, was shown to improve oxygen delivery to the exercising muscle, as evidenced by the training‐induced increase in oxygenated and total Hb at the end of endurance test to failure (Kacin & Strazar, 2011). However, Kacin and Strazar (2011) matched the total exercise volume for the two legs and found that the number of repetitions during the endurance test was improved more in the BFR leg than in the FBF leg. Our results are in contrast to theirs, highlighting the importance of exercise volume on the improvement in muscle endurance capacity via increased vasodilatation and/or capillary supply to the muscles.

The elbow flexion training with BFR and FBF caused a similar improvement in half‐time (i.e., the estimated time for peak torque output to decay by 50% during repetitive MVCs; Figure 7a). In contrast to the submaximal contractions used for determining *R*_max_, the determination of half‐time involved a series of MVCs, where full recruitment of motor units, including high‐threshold ones, could be assumed from the beginning to the end. Indeed, the biceps brachii muscle activity remained at a high level, despite the decrease in torque (Figure 1). The relative magnitude of muscle activity averaged over 60 contractions was similar between BFR and FBF and did not change significantly with training. These results suggest that the training‐induced improvement in half‐time mainly reflects muscular rather than neural adaptation and is not affected by the application of BFR.

Unlike *R*_max_, no significant association was found between the improvement in half‐time and the total exercise volume (Figure 7b). It is not surprising that *R*_max_ and half‐time showed different adaptations to training, because the motor unit recruitment during the measurement and thus the physiological determinant of performance would be considerably different between them. The lack of a favourable effect of BFR on half‐time appears to be contradictory to previous reports suggesting that resistance training with BFR induced a greater improvement in maintenance of maximal voluntary torque production than that without BFR in matched (Takarada et al., 2002) and unmatched (Pignanelli et al., 2020) exercise volumes. However, we found that BFR induced a greater improvement in half‐time than did FBF in seven of the eight participants (Figure 7a). Further study with a larger number of participants is warranted to clarify such individual variation in the effect of BFR on half‐time and its physiological mechanisms.

We showed that the training with BFR and FBF increased the muscle strength and thickness similarly (Figure 8). These results imply that low‐load resistance training with BFR is cost‐effective for inducing strength and hypertrophic adaptations, given that the total exercise volume was much smaller in BFR than in FBF. In fact, similar results have been reported by two recent studies (Farup et al., 2015; Pignanelli et al., 2020), suggesting that the magnitude of strength gains and muscle hypertrophy following low‐load resistance training depends not on the exercise volume itself, but on whether the exercise is performed to failure. Furthermore, our results raise the possibility that performing only one set to failure is a time‐efficient alternative to a traditional multiple‐set protocol of resistance exercise with BFR (Patterson et al., 2019), because the total exercise duration (including rest between sets) is shorter in the current protocol (typically 2–3 min) than in the traditional protocol (typically 4–5 min), while the number of repetitions is similar between the two. The relative importance of performing exercise to failure over the number of repetitions contrasts with the major finding of this study that increasing the total exercise volume would be important for improving the capacity of maintaining force production during submaximal contractions. We also found that the training‐induced improvement in 1RM but not in isometric strength was positively associated with the change in muscle thickness. In addition, there was no significant association between the changes in 1RM and isometric strengths. These results are in line with the principle of specificity in exercise training (Spitz et al., 2023) and might be responsible, in part, for the different changes in *R*_max_ (assessed with submaximal dynamic contractions) and half‐time (assessed with maximal isometric contractions) with training. In addition, the disproportionate increase in muscle strength relative to muscle thickness is well documented in previous resistance‐training studies and could be attributed mainly to neural adaptations (Škarabot et al., 2021) rather than to a change in muscle quality, because we found no significant change in echo intensity post‐intervention.

### 4.1 Limitations

In the present study, the participants were limited to healthy men, whose characteristics differed to some extent between experiments 1 and 2. Despite the differences in age, body mass and training experience, the reduction in the number of repetitions attributable to BFR was consistent in both experiments, which has also been reported in previous studies including both sexes (Ganesan et al., 2015; Kolind et al., 2023; Wernbom et al., 2009). Additionally, although we could detect a significant impact of BFR on the exercise volume and the post‐training improvement in *R*_max_, the small sample size in this study might prevent ‘less than a large effect’ from reaching statistical significance. Another limitation is the use of ultrasound‐based muscle thickness measurement, which is incapable of detecting three‐dimensional changes in muscle size with resistance training (Yasuda et al., 2012). Finally, and most importantly, further research is needed to determine whether our findings can be applied to multi‐joint and/or lower‐limb exercises. Nevertheless, recent studies using single‐leg squat (Petrick et al., 2019) and knee extension (Kolind et al., 2023) have shown that the application of BFR caused a reduction in the total number of repetitions, the magnitude of which was similar to that in the present study. We thus believe that the acute and chronic effects of BFR on muscle endurance performance and adaptations are consistent across types of exercises.

## 5 CONCLUSIONS

We showed that the improvement in muscle endurance performance of elbow flexors after 6 weeks of training was attenuated by the application of BFR during submaximal but not maximal contractions. In contrast, training with and without BFR resulted in similar improvements in muscle strength and thickness. These results suggest that the reduction in exercise volume can be a key factor in the negative impact of BFR on muscle endurance adaptation to low‐load resistance training.

## AUTHOR CONTRIBUTIONS

Conceptualization and methodology: Akito Ida, Kazushige Sasaki; Data curation, formal analysis and investigation: Akito Ida; Software: Akito Ida, Kazushige Sasaki; Writing—original draft preparation: Akito Ida; Writing—review and editing: Kazushige Sasaki; Visualization: Akito Ida; Funding acquisition and Supervision: Kazushige Sasaki.

## CONFLICT OF INTEREST

The authors declare no conflicts of interest.

## Data Availability

All data supporting the results in this paper are available as Supporting information.
